# Effects of an experimental short-time high-intensity warm-up on explosive muscle strength performance in soccer players: A pilot study

**DOI:** 10.3389/fphys.2022.984305

**Published:** 2022-08-25

**Authors:** Antonino Patti, Valerio Giustino, Norikazu Hirose, Giuseppe Messina, Stefania Cataldi, Giuseppe Grigoli, Alida Marchese, Giuseppe Mulè, Patrik Drid, Antonio Palma, Antonino Bianco

**Affiliations:** ^1^ Sport and Exercise Sciences Research Unit, Department of Psychology, Educational Science and Human Movement, University of Palermo, Palermo, Italy; ^2^ Faculty of Sport Sciences, Waseda University, Saitama, Japan; ^3^ Department of Basic Medical Sciences, Neuroscience and Sense Organs, University of Study of Bari, Bari, Italy; ^4^ Faculty of Sport and Physical Education, University of Novi Sad, Novi Sad, Serbia

**Keywords:** sport performance, soccer, warm-up, handgrip strength, jumping performance, strength, vertical jump height

## Abstract

**Objective:** This study aimed to evaluate the effects of an experimental short-time warm-up consisting of a small number of intermittent high-intensity sprints on explosive muscle strength performance in soccer players and to identify recovery times after performing the sprints. Furthermore, we evaluated the reliability of a smartphone app in jumping performance.

**Methods:** Twenty male soccer players were given the following tests: 1) the counter-movement jump (CMJ) test with the Microgate system, 2) the counter-movement jump (CMJ) test with the MyJump smartphone app, and 3) the handgrip strength test. The experimental short-time high-intensity warm-up was carried out 1 week after test administration. The warm-up consisted of three maximum sprints over 60 m with 120 s of recovery between sprints. Then, the tests were administered again: the vertical jump height (VJH) performances (five trials) were measured 90 s after the last sprint; the handgrip strength performances (three trials) were measured 120 s after the last vertical jump test.

**Results:** The maximum VJH was found in the third trial of the CMJ test, 330 s after the last sprint (*p* < 0.01), the result closest to the baseline. The lowest VJH was found in the first trial of the CMJ test, 90 s after the last sprint (*p* < 0.05). Pearson’s analysis between the CMJ test with the Microgate system and the CMJ test with MyJump showed a strong correlation (R = 0.96). Lin’s concordance correlation coefficient showed a substantial concordance (ρc = 0.959) between measures.

**Conclusion:** This experimental short-time warm-up of high-intensity intermittent sprints appears to be a simple, quick, and efficient activity to accelerate soccer players’ optimal performance.

## 1 Introduction

In soccer, coaches and athletic trainers emphasize on developing physical abilities such as explosive strength, endurance, and speed. The reason is related to the physical demands that soccer requires, such as explosive performance in sprint or vertical jump and endurance in running (Metaxas et al., 2005). For this reason, coaches and athletic trainers organize training loads based on physical demands and related energy expenditure. In fact, soccer players have to adapt to the demands of soccer, that is, to the stress of the anaerobic metabolism (Drust et al., 2000; Kunz et al., 2019).

It is well known that both a pre-match warm-up for the starting team and a half-time warm-up for substitute players are indispensable to preparing and to obtain the best possible performance during the match (McCrary et al., 2015; Silva et al., 2018; Patti et al., 2022). However, the time between the pre-match warm-up and the start of the match can adversely affect performance. Considering the short time available between the first and the second time, it is advisable to administer a suitable warm-up (Hammami et al., 2018). Hills et al. (2021) questioned the effectiveness of practices typically used by substitute players before pitch-entry. Mohr et al. (2004) showed that soccer players perform less high-intensity sprint at the initial phase of the second half compared with the first half. On the contrary, substitute players often have a short time available before pitch-entry (Hills et al., 2020). Hills et al. (2020) investigated the use and practices of substitutes in professional soccer. The authors showed that there is no certainty about the effectiveness of the current pre-pitch-entry practices, and the 100% of practitioners highlighted the need for further studies (Hills et al., 2020).

Mohr et al. (2004) demonstrated the positive effects of warm-up in muscles for sprint performance, but the effects on performance depend on the intensity of the activities. Different studies showed reduced physical performance after the half-time, and some authors explain this event as the lack of an adequate warm-up for muscles and temperature-related mechanisms demanded by the intensity of the activities and the short time available in the interval between the first and the second time (Bishop, 2003a; Bishop, 2003b; Mohr et al., 2004).

A high-intensity warm-up has been revealed to increase anaerobic energy provision, improving anaerobic metabolism, and these physiological mechanisms can result in improvement of the performance (Bishop, 2003a; Bishop, 2003b; McGowan et al., 2015; Yanaoka et al., 2020a; Yanaoka et al., 2020b). Intermittent high-intensity exercise such as repeated sprinting within relatively short time intervals is considered a relevant fitness prerequisite in competitive soccer players (Krustrup et al., 2006). Intermittent high-intensity sprints are defined as sprints of ≤10 s with recovery periods of >60–300 s between sprints. Intermittent high-intensity sprints can be an excellent strategy to reach an optimal muscle temperature before performance. This warm-up can be useful after the half-time warm-up in substitute players. However, intermittent high-intensity sprints can lead to decreased performance and neuromuscular fatigue (Balsom et al., 1992; Bishop and Claudius, 2005; Pearcey et al., 2016). Yanaoka et al. (2020b) suggested that an intermittent sprint is a short sprint interspersed with a recovery period long enough to allow for an almost complete recovery of sprint performance (Yanaoka et al., 2020a). Consequently, increasing the recovery time between sprints is a potential method for reducing neuromuscular fatigue and improving performance (Monks et al., 2017). For example, in a recent study, the athletes completed an intermittent sprint protocol that consisted of 10 s of rest, 5 s of maximum sprint, and 105 s of active recovery with repeated cycles over 10 min (Yanaoka et al., 2020a).

Furthermore, from the perspective of performance evaluation in sports, the measurement of jumping performance is of fundamental importance (Griffiths et al., 2019; Katushabe and Kramer, 2020; Giustino et al., 2022), especially through the use of dedicated devices. Recently, a smartphone-based application for jumping performance evaluation, named MyJump, has been widely used in various sports contexts. However, the validity and the reliability of this app are still unclear (Montalvo et al., 2021).

Based on this background, the first aim of this study was to evaluate the effects of an experimental short-time warm-up consisting of a small number of intermittent high-intensity sprints on explosive muscle strength performance in soccer players and to identify recovery times after performing the sprints. In addition, this study aimed to investigate the reliability of a smartphone app in jumping performance.

## 2 Materials and methods

### 2.1 Study design

The design of this pilot study was a “pre-post study” in which participants were assessed at two different times (T0 and T1). A total of 27 participants were enrolled; however, seven of them dropped out. Then, 20 male soccer players (age: 21.35 ± 4.79 years; weight: 70.75 ± 8.66 kg; height: 1.74 ± 0.05 m) of an Italian soccer team were included in the study. To be eligible for the study, participants had to be male with at least 3 years of soccer experience. Participants who had had any musculoskeletal injury in the past year could not be included in the study. Goalkeepers, due to their peculiar physical demands and the specific training they usually perform, were not included in the study.

Vertical jump height (VJH) performance and handgrip strength performance of participants were assessed at the baseline (T0) and immediately following an experimental short-time high-intensity warm-up performed 1 week after the baseline (T1). The experimental short-time high-intensity warm-up was carried out 1 week after baseline assessment in order to ensure the total neuromuscular recovery of participants. In the 24 h, prior to both test sessions (i.e., T0 and T1), all participants had not performed exhaustion exercises. Both test sessions were administered at the same time (between 4:00 and 6:00 p.m.).

The study was carried out in compliance with the principles of the Declaration of Helsinki and approved by the Ethics Committee of the Faculty of Sport and Physical Education of the University of Novi Sad (No. 46-10-02/2). Written informed consent was obtained from each participant before the participation in the study.

### 2.2 Study protocol and experimental short-time high-intensity warm-up

**FIGURE 1:**
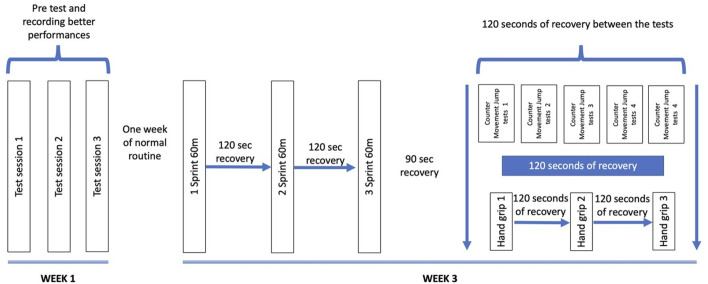
Study protocol and experimental short-time high-intensity warm-up.

As shown in Figure 1, the study protocol and the experimental short-time high-intensity warm-up consisted of 1) 5 min of general activation; 2) three maximum sprints on 60 m with 120 s of recovery between sprints; 3) 90 s of recovery; 4) five counter-movement jump (CMJ) tests with 120 s of recovery between the trials (R1, R2, R3, R4, and R5); 5) 120 s of recovery; and 6) three handgrip strength tests with 120 s of recovery between trials.

All measurements were carried out by a technician of the Italian Football Federation (FIGC) at the baseline (T0) and after the experimental warm-up (T1).

### 2.3 Measurements

At the baseline (T0), before administering the tests, participants were measured for their weight and height. Weight was evaluated using a scale to the nearest 100 g (WUNDER 960 Classic; Trezzo sull’Adda, Milano, Italia). Height was evaluated using a stadiometer sensitive to changes of 1 cm (Seca 220; Hamburg, Germany).

The tests administered both at the baseline (T0) and after 1 week (T1) were as follows: 1) the counter-movement jump (CMJ) test with the Microgate system (Montalvo et al., 2021); 2) the counter-movement jump (CMJ) test with MyJump smartphone app (Stanton et al., 2017); and 3) the handgrip strength test (Bianco et al., 2016; Battaglia et al., 2018). The best performance of each test was considered in order to have the participants’ best performances by creating a baseline.

### 2.3.1 Counter-movement jump test with the Microgate system

It is an optical detection system composed of a transmitting and a receiving bar. The system allows the measurement of flight and contact time of a jump with a precision of 1/1,000 of a second. Starting from these fundamental basic data, the dedicated software allows to obtain a series of parameters related to jumping performance with maximum precision and in real-time (Montalvo et al., 2021).

#### 2.3.2 Counter-movement jump test with MyJump smartphone app

It is a smartphone app that allows to record jumping performance. Each CMJ was recorded in the sagittal plane with 240 Hz sampling. The choice to use the sagittal plane was made according to the protocol by Stanton et al. (2017). Indeed, the authors showed that the detection of the jump was more precise in the sagittal plane than in the frontal plane. The iPhone (Apple Inc., Cupertino, CA) was inserted in a support, and the video recording was started using a Bluetooth remote control, enabling the camera to remain in an identical, static position for all CMJ trials. Video collected by the iPhone was processed using the MyJump smartphone app (Stanton et al., 2017).

#### 2.3.3 Handgrip strength test

This test aims to measure the maximum isometric strength exerted by the muscles of the hand, in particular, the muscles responsible for flexion of the metatarsi and phalanges, flexion of the fingers, and adduction of the thumb (Bianco et al., 2016; Patti et al., 2017; Garcia-Pinillos et al., 2018; Bonaventura et al., 2020).

### 2.4 Statistical analysis

All data were recorded in an Excel file. We performed *a priori* power analysis to calculate the sample size required using a power of 0.80 (G*Power software version 3.1.9.2; Heinrich Heine University, Düsseldorf, Germany). Then, we calculated a *post hoc* power analysis to determine the power of the sample size recruited. The Shapiro–Wilk normality test was used to analyze data distribution. Repeated-measures analysis of variance and the Tukey test were used for comparisons. Pearson’s analysis was used to evaluate the performance correlation between the CMJ test with the Microgate system and the CMJ test with MyJump smartphone app. These analyses were conducted *via* Jamovi software (version 2.3.0.0, The jamovi project (2021)) with the significance level set at 0.05. Furthermore, Lin’s concordance correlation coefficient between the CMJ test with the Microgate system and the CMJ test with MyJump was used to measure the reliability of the smartphone app. This statistical test was carried out by MedCalc software (version 20.112, MedCalc Software Ltd, Ostend, Belgium).

## 3 Results

*A priori* sample size power analysis required a number of 27 participants. However, since seven participants dropped out, only 20 participants were eligible for the study. Hence, a *post hoc* sample size analysis revealed a power of 70%.

**FIGURE 2:**
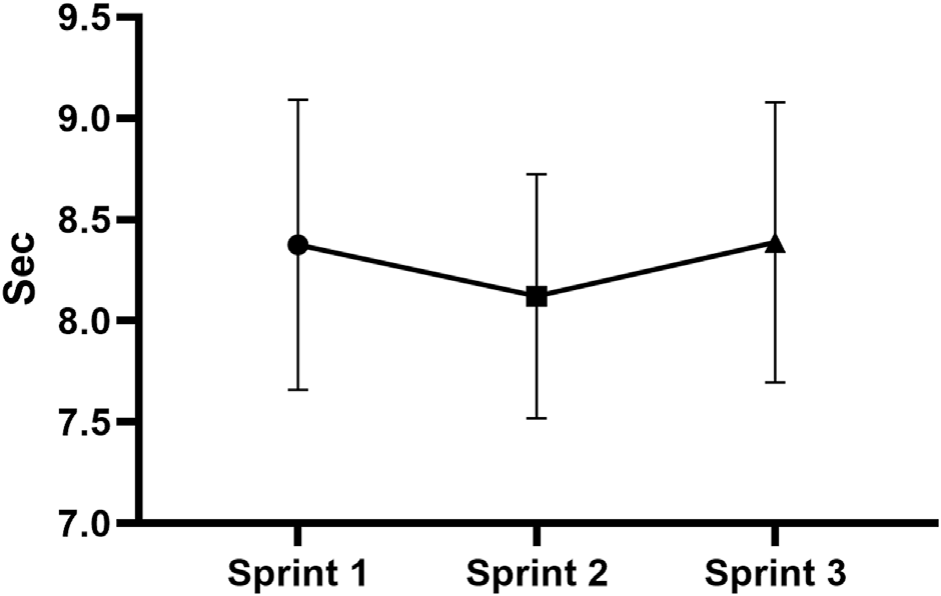
Chronometric measures of sprints.

The chronometric measures of sprints have been maintained without significant differences (Figure 2).

The Shapiro–Wilk normality test showed a Gaussian distribution of variables.

**FIGURE 3:**
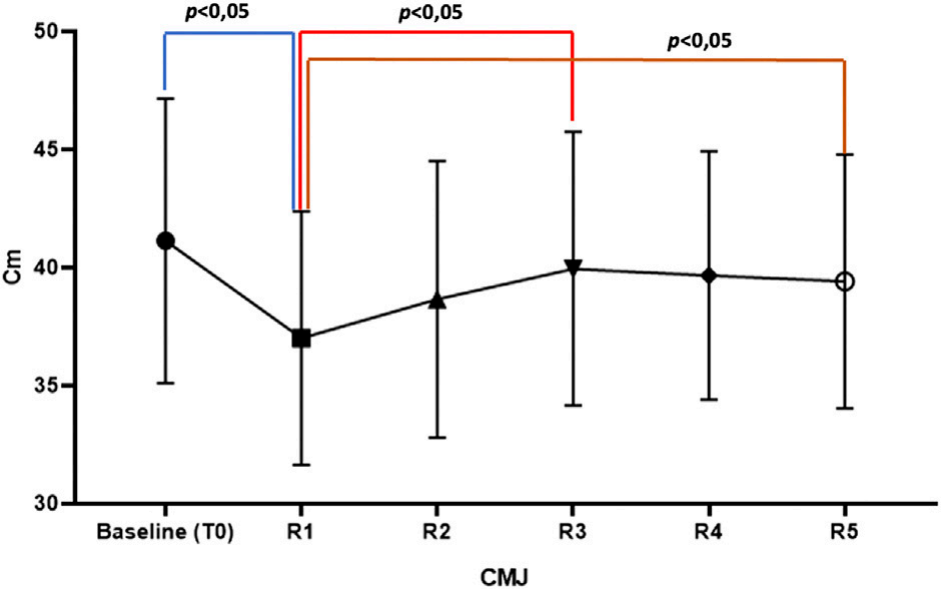
CMJ performances.

As reported in Table 1, the maximum VJH was found in the third trial (R3) of the CMJ test, 330 s after the last sprint (R1 vs. R3, *p* < 0.01), the result closest to the baseline. The lowest VJH was found in the first trial (R1) of the CMJ test, 90 s after the last sprint (*p* < 0.05). Table 2 shows the results of repeated-measures analysis of variance and the Tukey test. Figure 3 shows the performances of the CMJ test at T0 and of all the trials at T1. The handgrip strength test showed no significant changes after the experimental short-time high-intensity warm-up.

**TABLE 1:** Descriptive analysis of CMJ performances with the Microgate system.

	T0	T1				
	Baseline	R1	R2	R3	R4	R5
N	20	20	20	20	20	20
Mean	41.1	37.0	38.7	39.9	39.7	39.4
Median	40.3	37.6	39.7	39.8	39.4	39.7
Standard deviation	6.02	5.37	5.85	5.80	5.27	5.37

**TABLE 2:** CMJ performances with the Microgate system—*post hoc* comparisons vs. time.

Comparison		Mean difference	df	t	*p*
Time	Time				
T0	R1	4.125	19	3.168	*p* < 0.05
	R2	2.475	19	2.095	ns
	R3	1.190	19	0.967	ns
	R4	1.470	19	1.355	ns
	R5	1.720	19	1.552	ns
R1	R2	−1.650	19	−2.474	ns
	R3	−2.935	19	−3.767	*p* < 0.05
	R4	−2.655	19	−3.029	ns
	R5	−2.405	19	−3.346	*p* < 0.05
R2	R3	−1.285	19	−2.209	ns
	R4	−1.005	19	−1.349	ns
	R5	−0.755	19	−1.210	ns
R3	R4	0.280	19	0.625	ns
	R5	0.530	19	0.964	ns
R4	R5	0.250	19	0.551	ns

**FIGURE 4:**
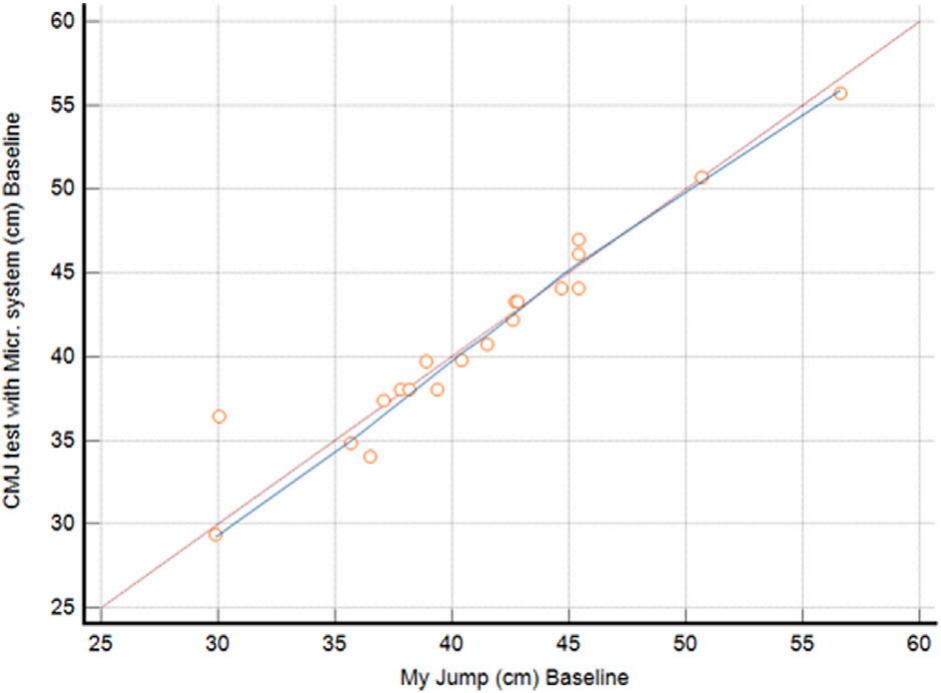
Concordance correlation coefficient of CMJ performances.

Pearson’s analysis between the CMJ test with the Microgate system at T0 and the CMJ test with MyJump smartphone app at T0 showed a strong correlation (R = 0.96). Furthermore, Lin’s concordance correlation coefficient showed a substantial concordance (ρc = 0.959) between measures, as shown in Table 3 and Figure 4. Hence, these results show a similar trend between the CMJ tests measured with the two different systems (Tables 4, 5).

**TABLE 3:** CMJ performance comparison between the Microgate system and MyJump smartphone app.

Microgate system vs. MyJump smartphone app		Mean difference	df	p	Pearson’s correlation coefficient (R)	Lin’s concordance correlation coefficient (ρc)
Microgate system (cm) T0	MyJump (cm) T0	0.0485	19	0.903	0.960	0.959
Microgate system (cm) R1	MyJump (cm) R1	0.4350	19	0.027	0.988	0.985
Microgate system (cm) R2	MyJump (cm) R2	0.2300	19	0.115	0.994	0.993
Microgate system (cm) R3	MyJump (cm) R3	0.2000	19	0.193	0.993	0.993
Microgate system (cm) R4	MyJump (cm) R4	0.1050	19	0.559	0.991	0.989
Microgate system (cm) R5	MyJump (cm) R5	0.0350	19	0.877	0.982	0.981

**TABLE 4:** Descriptive analysis of CMJ performance with MyJump.

	T0	T1				
	Baseline	R1	R2	R3	R4	R5
N	20	20	20	20	20	20
Mean	41.1	36.6	38.4	39.7	39.6	39.4
Median	41.0	37.3	39.4	39.7	39.7	39.3
Standard deviation	6.28	5.33	5.87	5.94	5.58	5.15

**TABLE 5:** CMJ performances with MyJump app—*post hoc* comparisons vs. time.

Comparison		Mean difference	df	t	*p*
Time	Time				
T0	R1	4.511	19	3.382	*p* < 0.05
	R2	2.656	19	2.042	ns
	R3	1.341	19	1.031	ns
	R4	1.526	19	1.319	ns
	R5	1.707	19	1.406	ns
R1	R2	−1.855	19	−2.848	ns
	R3	−3.170	19	−4.415	*p* < 0.01
	R4	−2.985	19	−3.325	*p* < 0.05
	R5	−2.805	19	−3.622	*p* < 0.05
R2	R3	−1.315	19	−2.289	ns
	R4	−1.130	19	−1.600	ns
	R5	−0.950	19	−1.448	ns
R3	R4	0.185	19	0.352	ns
	R5	0.365	19	0.567	ns
R4	R5	0.180	19	0.353	ns

## 4 Discussion

The first aim of this study was to evaluate the effects of an experimental short-time warm-up consisting of a small number of intermittent high-intensity sprints on explosive muscle strength performance in soccer players and to identify recovery times after performing the sprints. Furthermore, this study aimed to investigate the reliability of a smartphone app in jumping performance.

First, to date, it is not entirely clear whether a warm-up with intermittent high-intensity exercises facilitates performance. Our aim was to maintain the same high-intensity exercise during the sprints. Indeed, the sprint chronometric measurements were maintained without significant differences. Therefore, both the number of sprints and the recovery times adopted were supported by the analysis of the sprint times. In this study, we administered three maximum sprints on 60 m with 120 s of recovery between the sprints. Few studies in the literature have shown positive results on sprints. For instance, Mohr et al. (2004) reported a significantly improved repeated sprint time using three maximum sprints on 30 m with 25 s of recovery.

The results showed the positive effects of the experimental short-time high-intensity warm-up on explosive muscle strength performance in soccer players. In fact, the intermittent high-intensity sprints did not affect the CMJ performances. In detail, the results showed that the performance of CMJ performed immediately after the experimental short-time high-intensity warm-up (R1) was significantly worse than the baseline. Consequently, these results suggested that 90 s after the last sprint, participants had not yet recovered optimal anaerobic capacity. Our data indicate that only 330 s after the last sprint, the performance of CMJ overlaps the baseline (R3). These results are in line with the findings of a previous meta-analysis in which the authors showed that performance was optimal after repeated exercises performed at intensities between 60 and 84% and using rest periods between 7 and 10 min (Wilson et al., 2013). Smith and Fry (2007) demonstrated that the regulatory myosin light-chain (RLC) phosphorylation of positive responders had increased 23% at 5 min of recovery. The literature suggests that the RLC is the primary mechanism responsible for post-activation potentiation (PAP) and improves the interaction between myosin and actin filaments (Grange et al., 1993; Sweeney et al., 1993). Mohr et al. (2004) demonstrated that muscle temperature affects athletes’ performance. Lower performance was associated with a decrease in muscle and core temperature. The warm-up should improve acute explosive performance, especially for athletes who perform many intermittent sprints during the match (Edge et al., 2005).

As for the duration of the warm-up, a previous research compared the effects of a long warm-up (general + specific) and a short warm-up (specific) on intermediate running performance (3-minute run) (van den Tillaar et al., 2017). Since no significant differences in running performance and physiological parameters were found between the two warm-up protocols, authors stated that a short warm-up is as effective as a long warm-up for intermediate running performance (van den Tillaar et al., 2017).

Regarding the recovery times, the literature suggests that a passive rest period reduces performance. Our data showed that a short-time warm-up consisting of intermittent high-intensity sprints could bring athletes’ performance to optimal levels after 330 s of recovery. These findings are in line with the results shown by other research groups in which the authors described that running at 70% HRmax (Zois et al., 2013) and after a series of jumps improved performance compared to traditional rest (Abade et al., 2017).

The handgrip strength performances showed no significant change after 690 s of recovery from the last sprint. Some studies have described that the presence/absence of supraspinal fatigue (or central fatigue) can be determined by changes in strength muscle performance (e.g., handgrip strength) (Paavolainen et al., 1999; Kilduff et al., 2007; Martin et al., 2010). Based on our handgrip strength results, we can suppose that our sample showed no central fatigue after the experimental warm-up.

Furthermore, the results of the CMJ using MyJump smartphone app showed a strong correlation with the values obtained with the Microgate system. These findings confirm the reliability of the application, which can be used for easier data collection for future studies.

## 5 Conclusion

In conclusion, this study highlights that a short-time high-intensity warm-up composed of intermittent sprints appears to be a simple, quick, and efficient activity to accelerate soccer players’ optimal performance. This experimental warm-up is of short duration in order to be considered applicable also between the first and the second time. For this reason, exercises and recovery times have been selected so that the entire protocol can also be administered during the interval of a match. Our data show that this short-time warm-up can be administered in the interval between the first and second time. In fact, our results indicate that a high-intensity warm-up in the short-time interval can ensure physical conditions necessary for high-performance levels during the second half time. A previous study investigated the effects of different half-time re-warm-up durations on intermittent sprint performance, finding that both the 3-min re-warm-up and the 7-min re-warm-up were more effective in improving intermittent sprint performance after the half-time than a traditional passive half-time practice (Yanaoka et al., 2018). However, further studies are needed to confirm our results based on the fact that some studies have shown that the warm-up is a complex question since volume changes, intensity, and recovery may negatively affect the subsequent performance.

Finally, among the limitations to this study, it should be mentioned that the sample size did not reach the minimum power of 80% required, but it was slightly lower (70%).

## Data Availability

The raw data of this article will be made available by the authors, without undue reservation.

## References

[B1] Abade E. Sampaio J. Goncalves B. Baptista J. Alves A. Viana J. (2017). Effects of different Re-warm up activities in Football players’ performance. Plos One 12, E0180152. 10.1371/journal.pone.0180152 28662123 PMC5491134

[B2] Balsom P. D. Seger J. Y. Sjodin B. Ekblom B. (1992). Physiological responses to maximal intensity intermittent exercise. Eur. J. Appl. Physiol. Occup. Physiol. 65, 144–149. 10.1007/BF00705072 1396638

[B3] Battaglia G. Messina G. Giustino V. Zangla D. Barcellona M. Iovane A. (2018). Influence of vertical dimension of occlusion on peak force during handgrip tests in athletes. Asian J. Sports Med. 9, E68274. 10.5812/asjsm.68274

[B4] Bianco A. Mammina C. Jemni M. Filippi A. R. Patti A. Thomas E. (2016). A fitness index model for Italian adolescents living in southern Italy: The asso project. J. Sports Med. Phys. Fit. 56, 1279–1288. 26472604

[B5] Bishop D. Claudius B. (2005). Effects of induced metabolic alkalosis on prolonged intermittent-sprint performance. Med. Sci. Sports Exerc. 37, 759–767. 10.1249/01.mss.0000161803.44656.3c 15870629

[B6] Bishop D. (2003a). Warm up I: Potential mechanisms and the effects of passive warm up on exercise performance. Sports Med. 33, 439–454. 10.2165/00007256-200333060-00005 12744717

[B7] Bishop D. (2003b). Warm up ii: Performance changes following active warm up and how to structure the warm up. Sports Med. 33, 483–498. 10.2165/00007256-200333070-00002 12762825

[B8] Bonaventura R. E. Giustino V. Chiaramonte G. Giustiniani A. Smirni D. Battaglia G. (2020). Investigating prismatic adaptation effects in handgrip strength and in plantar pressure in healthy subjects. Gait Posture 76, 264–269. 10.1016/j.gaitpost.2019.12.022 31881480

[B9] Drust B. Cable N. T. Reilly T. (2000). Investigation of the effects of the pre-cooling on the physiological responses to soccer-specific intermittent exercise. Eur. J. Appl. Physiol. 81, 11–17. 10.1007/PL00013782 10552261

[B10] Edge J. Bishop D. Goodman C. Dawson B. (2005). Effects of high- and moderate-intensity training on metabolism and repeated sprints. Med. Sci. Sports Exerc. 37, 1975–1982. 10.1249/01.mss.0000175855.35403.4c 16286869

[B11] Garcia-Pinillos F. Delgado-Floody P. Martinez-Salazar C. Latorre-Roman P. A. (2018). Responsiveness of the countermovement jump and handgrip strength to an incremental running test in endurance athletes: Influence of sex. J. Hum. Kinet. 61, 199–208. 10.1515/hukin-2017-0121 29599872 PMC5873349

[B12] Giustino V. Messina G. Patti A. Padua E. Zangla D. Drid P. (2022). Effects of A postural exercise program on vertical jump height in young female volleyball players with knee valgus. Int. J. Environ. Res. Public Health 19, 3953. 10.3390/ijerph19073953 35409635 PMC8997520

[B13] Grange R. W. Vandenboom R. Houston M. E. (1993). Physiological significance of myosin phosphorylation in skeletal muscle. Can. J. Appl. Physiol. 18, 229–242. 10.1139/h93-020 8242003

[B14] Griffiths B. Grant J. Langdown L. Gentil P. Fisher J. Steele J. (2019). The effect of in-season traditional and explosive resistance training programs on strength, jump height, and speed in recreational soccer players. Res. Q. Exerc. Sport 90, 95–102. 10.1080/02701367.2018.1563276 30707090

[B15] Hammami A. Zois J. Slimani M. Russel M. Bouhlel E. (2018). The efficacy and characteristics of warm-up and Re-Warm-Up practices in soccer players: A systematic review. J. Sports Med. Phys. Fit. 58 (1-2), 135–149. 10.23736/S0022-4707.16.06806-7 27901341

[B16] Hills S. P. Aben H. G. J. Starr D. P. Kilduff L. P. Arent S. M. Barwood M. J. (2021). Body temperature and physical performance responses are not maintained at the time of pitch-entry when typical substitute-specific match-day practices are adopted before simulated soccer match-play. J. Sci. Med. Sport 24, 511–516. 10.1016/j.jsams.2020.11.013 33317982

[B17] Hills S. P. Radcliffe J. N. Barwood M. J. Arent S. M. Cooke C. B. Russell M. (2020). Practitioner perceptions regarding the practices of soccer substitutes. Plos One 15, E0228790. 10.1371/journal.pone.0228790 32032369 PMC7006909

[B18] Katushabe E. T. Kramer M. (2020). Effects of combined power band resistance training on sprint speed, agility, vertical jump height, and strength in collegiate soccer players. Int. J. Exerc. Sci. 13, 950–963. 32922637 10.70252/WZLX5662 PMC7449328

[B19] Kilduff L. P. Bevan H. R. Kingsley M. I. Owen N. J. Bennett M. A. Bunce P. J. (2007). Postactivation potentiation in professional rugby players: Optimal recovery. J. Strength Cond. Res. 21, 1134–1138. 10.1519/R-20996.1 18076243

[B20] Krustrup P. Mohr M. Steensberg A. Bencke J. Kjaer M. Bangsbo J. (2006). Muscle and blood metabolites during A soccer game: Implications for sprint performance. Med. Sci. Sports Exerc. 38, 1165–1174. 10.1249/01.mss.0000222845.89262.cd 16775559

[B21] Kunz P. Engel F. A. Holmberg H. C. Sperlich B. (2019). A meta-comparison of the effects of high-intensity interval training to those of small-sided games and other training protocols on parameters related to the Physiology and performance of youth soccer players. Sports Med. Open 5, 7. 10.1186/s40798-019-0180-5 30790134 PMC6384288

[B22] Martin V. Kerherve H. Messonnier L. A. Banfi J. C. Geyssant A. Bonnefoy R. (2010). Central and peripheral contributions to neuromuscular fatigue induced by A 24-H treadmill run. J. Appl. Physiol. 108, 1224–1233. 10.1152/japplphysiol.01202.2009 20167672

[B23] Mccrary J. M. Ackermann B. J. Halaki M. (2015). A systematic review of the effects of upper body warm-up on performance and injury. Br. J. Sports Med. 49, 935–942. 10.1136/bjsports-2014-094228 25694615

[B24] Mcgowan C. J. Pyne D. B. Thompson K. G. Rattray B. (2015). Warm-up strategies for sport and exercise: Mechanisms and applications. Sports Med. 45, 1523–1546. 10.1007/s40279-015-0376-x 26400696

[B25] Metaxas T. I. Koutlianos N. A. Kouidi E. J. Deligiannis A. P. (2005). Comparative study of field and laboratory tests for the evaluation of aerobic capacity in soccer players. J. Strength Cond. Res. 19, 79–84. 10.1519/14713.1 15707383

[B26] Mohr M. Krustrup P. Nybo L. Nielsen J. J. Bangsbo J. (2004). Muscle temperature and sprint performance during soccer matches—beneficial effect of Re-Warm-Up at half-time. Scand. J. Med. Sci. Sports 14, 156–162. 10.1111/j.1600-0838.2004.00349.x 15144355

[B27] Monks M. R. Compton C. T. Yetman J. D. Power K. E. Button D. C. (2017). Repeated sprint ability but not neuromuscular fatigue is dependent on short versus long duration recovery time between sprints in healthy males. J. Sci. Med. Sport 20, 600–605. 10.1016/j.jsams.2016.10.008 27825551

[B28] Montalvo S. Gonzalez M. P. Dietze-Hermosa M. S. Eggleston J. D. Dorgo S. (2021). Common vertical jump and reactive strength index measuring devices: A validity and reliability analysis. J. Strength Cond. Res. 35, 1234–1243. 10.1519/JSC.0000000000003988 33629975

[B29] Paavolainen L. Nummela A. Rusko H. Hakkinen K. (1999). Neuromuscular characteristics and fatigue during 10 Km running. Int. J. Sports Med. 20, 516–521. 10.1055/s-1999-8837 10606214

[B30] Patti A. Giustino V. Cataldi S. Stoppa V. Ferrando F. Marvulli R. (2022). Effects of 5-week of fifa 11+ warm-up program on explosive strength, speed, and perception of physical exertion in elite female futsal athletes. Sports 10, 100. 10.3390/sports10070100 35878111 PMC9322867

[B31] Patti A. Maggio M. C. Corsello G. Messina G. Iovane A. Palma A. (2017). Evaluation of fitness and the balance levels of children with A diagnosis of juvenile idiopathic arthritis: A pilot study. Int. J. Environ. Res. Public Health 14, E806. 10.3390/ijerph14070806 28753965 PMC5551244

[B32] Pearcey G. E. Bradbury-Squires D. J. Monks M. Philpott D. Power K. E. Button D. C. (2016). Arm-cycling sprints induce neuromuscular fatigue of the elbow flexors and alter corticospinal excitability of the biceps brachii. Appl. Physiol. Nutr. Metab. 41, 199–209. 10.1139/apnm-2015-0438 26799694

[B33] Silva L. M. Neiva H. P. Marques M. C. Izquierdo M. Marinho D. A. (2018). Effects of warm-up, post-warm-up, and Re-Warm-Up strategies on explosive efforts in team sports: A systematic review. Sports Med. 48, 2285–2299. 10.1007/s40279-018-0958-5 29968230

[B34] Smith J. C. Fry A. C. (2007). Effects of A ten-second maximum voluntary contraction on regulatory myosin light-chain phosphorylation and dynamic performance measures. J. Strength Cond. Res. 21, 73–76. 10.1519/00124278-200702000-00014 17313284

[B35] Stanton R. Wintour S. A. Kean C. O. (2017). Validity and intra-rater reliability of MyJump app on iphone 6s in jump performance. J. Sci. Med. Sport 20, 518–523. 10.1016/j.jsams.2016.09.016 27876280

[B36] Sweeney H. L. Bowman B. F. Stull J. T. (1993). Myosin light chain phosphorylation in vertebrate striated muscle: Regulation and function. Am. J. Physiol. 264, C1085–C1095. 10.1152/ajpcell.1993.264.5.C1085 8388631

[B37] Van Den Tillaar R. Vatten T. Von Heimburg E. (2017). Effects of short or long warm-up on intermediate running performance. J. Strength Cond. Res. 31, 37–44. 10.1519/JSC.0000000000001489 27191697

[B38] Wilson J. M. Duncan N. M. Marin P. J. Brown L. E. Loenneke J. P. Wilson S. M. (2013). Meta-analysis of postactivation potentiation and power: Effects of conditioning activity, volume, gender, rest periods, and training status. J. Strength Cond. Res. 27, 854–859. 10.1519/JSC.0b013e31825c2bdb 22580978

[B39] Yanaoka T. Hamada Y. Fujihira K. Yamamoto R. Iwata R. Miyashita M. (2020a). High-intensity cycling Re-warm up within A very short time-frame increases the subsequent intermittent sprint performance. Eur. J. Sport Sci. 20, 1307–1317. 10.1080/17461391.2020.1713901 31914360

[B40] Yanaoka T. Iwata R. Yoshimura A. Hirose N. (2020b). A 1-minute Re-warm up at high-intensity improves sprint performance during the Loughborough intermittent shuttle test. Front. Physiol. 11, 616158. 10.3389/fphys.2020.616158 33519521 PMC7838537

[B41] Yanaoka T. Kashiwabara K. Masuda Y. Yamagami J. Kurata K. Takagi S. (2018). The effect of half-time Re-warm up duration on intermittent sprint performance. J. Sports Sci. Med. 17, 269–278. 29769828 PMC5950744

[B42] Zois J. Bishop D. Fairweather I. Ball K. Aughey R. J. (2013). High-Intensity Re-Warm-ups enhance soccer performance. Int. J. Sports Med. 34, 800–805. 10.1055/s-0032-1331197 23444096

